# Changes in self-perceived performance and satisfaction with performance of daily activities following interdisciplinary rehabilitation in people with late effects of polio

**DOI:** 10.3233/NRE-230219

**Published:** 2024-03-11

**Authors:** Katja Appelin, Lena-Karin Erlandsson, Jan Lexell, Eva Månsson Lexell

**Affiliations:** a Department of Neurology, Rehabilitation Medicine, Memory Disorders, and Geriatrics, Skåne University Hospital, Lund-Malmö, Sweden; b Department of Health Sciences, Lund University, Lund, Sweden; c School of Health and Welfare, Halmstad University, Halmstad, Sweden

**Keywords:** Occupational performance, outcome, post-poliomyelitis syndrome, daily activities, rehabilitation

## Abstract

**BACKGROUND:**

People with late effects of polio (LEoP) may need rehabilitation to manage everyday life but knowledge of the benefits of interdisciplinary rehabilitation is limited.

**OBJECTIVE:**

To evaluate changes in performance and satisfaction with performance of activities among people with LEoP following interdisciplinary rehabilitation.

**METHODS:**

A pre-post retrospective study based on data on 102 participants with LEoP from a rehabilitation clinic. Changes in performance and satisfaction with performance of daily activities before and after interdisciplinary rehabilitation were assessed with the Canadian Occupational Performance Measure (COPM).

**RESULTS:**

There were statistically significant increases in the mean performance and mean satisfaction with performance COPM scores from admission to discharge. Twenty-three percent and 19% of the participants, respectively, had improved their performance and satisfaction with performance, 25% and 26% of the participants had no changes, and 19% and 22% of the participants, respectively, rated their performance and satisfaction lower at discharge compared to admission.

**CONCLUSION:**

Interdisciplinary rehabilitation can enhance self-rated performance and satisfaction with performance of daily activities among people with LEoP. Future studies of rehabilitation for people with LEoP should use a prospective design and capture the participants’ process of change related to their rehabilitation period.

## 1 Introduction

Late effects of polio (LEoP) is a progressive neuromuscular condition that develops in up to 80% of people who in their childhood had paralytic polio (McNalley et al., 2015). The main impairments are new progressive muscle weakness, muscular fatigue, general fatigue, and pain (McNalley et al., 2015), and become more pronounced over time. It is also well-known that LEoP affects activity and participation (Ahlström & Karlsson, 2000; Appelin et al., 2014; Kling et al., 2002; Thorén-Jönsson, 2001), and many must find new ways to adapt their daily activities (Appelin et al., 2014; Jönsson et al., 1999; Sjödahl Hammarlund et al., 2020). There is no cure so access to individualized rehabilitation is essential (Lo & Robinson, 2018). It is important that rehabilitation not only focus on impairments but also include interventions where people learn how to adapt to the consequences that their LEoP may cause in their everyday lives (Jönsson et al., 1999).

The importance of incorporating a biomedical as well as a psychosocial model in rehabilitation is well-known, and rehabilitation is often described as a problem-solving educational process that requires interdisciplinary teamwork (Wade, 2015). The interdisciplinary teamwork is defined by coordinated interventions based on the different professionals’ knowledge and the person’s unique needs and goals. Professionals in interdisciplinary teams have regular meetings in order to discuss and collaboratively work towards the goals and together they carry out the interventions (Körner, 2010). Teamwork and team effectiveness are higher in teams working with the interdisciplinary team approach compared to a multidisciplinary team (Körner, 2010). The problem-solving educational process supports the individual to take an active role during goal setting and when interventions are implemented, and using an ICF-based rehabilitation plan during this process is therefore advantageous (Lexell & Brogårdh, 2015). When people with LEoP, together with professionals, actively participate in developing a mutually shared rehabilitation plan they gain a better understanding of their own participation during the rehabilitation process and goals focus on activity and participation to a greater extent (Månsson Lexell et al., 2015).

However, studies that have evaluated the outcome following interdisciplinary rehabilitation in people with LEoP are very few.

Qualitative studies (Larsson Lund & Lexell, 2010; Månsson Lexell et al., 2015) have shown that people with LEoP who participated in an individualized, goal-oriented comprehensive rehabilitation reached long-term positive benefits. Three studies (Bertelsen et al., 2009; Davidson et al., 2009; Curtis et al., 2020) have evaluated benefits of rehabilitation for people with LEoP. Bertelsen et al. (2009) reported better functional capacity and quality of life up to one year after a physiotherapy intervention performed within a multidisciplinary rehabilitation context. However, activity and participation were not their targeted outcome. The other two studies (Davidson et al., 2009; Curtis et al., 2020) assessed activity and participation in terms of performance and satisfaction with performance in daily activities, according to the Canadian Occupational Performance Measure (COPM) (Law et al., 2014). Davidson et al. (2009) evaluated if a nine-day multidisciplinary rehabilitation period could improve physical function, mood, activity, and participation among 27 people with LEoP. They reported significant improvements regarding exercise endurance, depression, and levels of fatigue. Five of 24 of the participants who responded to the COPM, improved their performance and satisfaction with performance of daily activties. Curtis et al. (2020) also used the COPM to evaluate activity and participation in 153 of 217 participants with LEoP who participated in a self-management group program. They reported significant improvements in satisfaction with performance of daily activities, whereas performance of daily activities had not changed. Despite being the only two studies that have used the COPM with participants with LEoP, the COPM is a commonly used tool to evaluate the benefits of rehabilitation in other populations (Sturkenboom et al., 2014; Townsend & Polatajko, 2007; Wressle et al., 2002; Månsson Lexell et al., 2014), by means of how participants perceive their changes in performance and satisfaction with performance. Changes include not only an increase in performance and satisfaction with performance among participants, but also those where performance and satisfaction with performance have decreased or not changed at all.

Taken together, there is limited knowledge of the benefits of interdisciplinary rehabilitation individually delivered to people with LeoP in terms of activity and participation, specifically how performance and satisfaction with performance of daily activities change after an interdisciplinary rehabilitation period. Thus, the objective of the present study was to evaluate changes in performance and satisfaction with performance of daily activities among people with LEoP following interdisciplinary rehabilitation.

## 2 Methods

### 2.1 Study design

This study has a pre-post design without a control group and is based on retrospective data from a database in a rehabilitation clinic at a University Hospital in Southern Sweden.

### 2.2 Participants

**Fig. 1:**
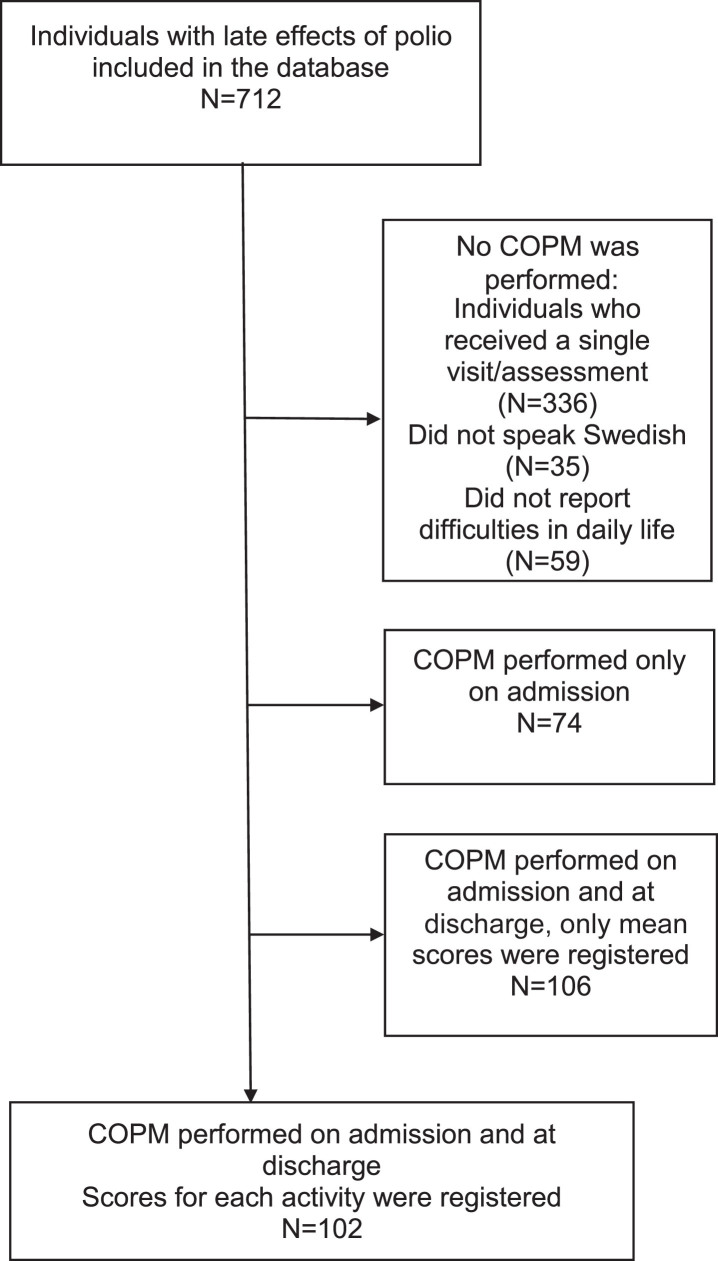
Flowchart showing how the participants were selected.

In Fig. 1, a flowchart of the participant inclusion is presented. Between 2004–2015, a total of 712 people with LEoP received different types of rehabilitation interventions according to each individual’s specific needs. That is, interventions varied from single interventions provided by single professionals to more comprehensive interventions provided by an interdisciplinary team, i.e., the interdisciplinary rehabilitation program. Data collected during their rehabilitation were registered in the database.

To be eligible for inclusion in the present study, the individuals should have been admitted to the interdisciplinary rehabilitation program between the years 2004 and 2015, be 18 years of age or older, have a confirmed history of polio and a clinically confirmed LEoP diagnosis, and have been assessed with the Canadian Occupational Performance Measure (COPM) (Law et al., 2014) before and after their rehabilitation period. Individuals who were not able to understand and speak Swedish or had cognitive impairments or other disease(-s) that would significantly impact their disability, were excluded. For 102 participants, COPM was performed on admission and at discharge and thereby comprised the final sample.

### 2.3 Ethical considerations

The study has been approved by the Swedish Ethical Review Authority (2020–05313) and was performed in accordance with medical research involving human subjects as described in the Declaration of Helsinki.

### 2.4 The interdisciplinary rehabilitation program

The main aim of the interdisciplinary rehabilitation program was to reduce self-perceived disability by providing a variety of interventions tailored to each individual (Larsson Lund & Lexell, 2010). The program used the principles of self-management theory (Lorig & Holman, 2003). The rehabilitation team consisted of a rehabilitation medicine physician, a physiotherapist, an occupational therapist, and a social worker. All team members had experience in interdisciplinary teamwork and rehabilitation of individuals with LEoP. The program followed a process that was built upon a four-step process comprising: i) assessment; ii) goal setting and planning; iii) implementing interventions and iv) evaluation (Lexell, 2012).

The rehabilitation program started with a visit to the clinic. The physician assessed the clinical symptoms, reviewed the medical history, and performed a neurological examination. Prior to the first visit, an EMG had been performed to verify prior polio. This information was also used as a guide to plan and implement correct interventions during the rehabilitation program. The other team members carried out general assessments according to an interdisciplinary checklist. The results from the assessments were discussed with the individual and summarized in a document, together with information about the planned interdisciplinary rehabilitation period.

On admission to the interdisciplinary rehabilitation program, extended assessments were performed by each team member. Building on our previous research (Lexell & Brogårdh, 2015; Månsson Lexell et al., 2015), an ICF-based rehabilitation plan was used as a mutual tool shared by the individual and the team members; the results from the extended assessments were discussed with the individual and summarized in the ICF-based rehabilitation plan. The assessments also served to increase each individual’s awareness of their problems, thereby facilitating a process of change. Thereafter, the individual and the team members together formulated goals that were relevant and tailored to each individual’s specific needs. As a result of the standardized procedure with individually set goals that took different times to achieve, the length of the rehabilitation also varied for each individual. Altogether, the rehabilitation period lasted approximately three months, where some (1–4 weeks) time was spent at the rehabilitation facility and the remaining at home.

During the interdisciplinary rehabilitation program, various interventions were implemented according to the ICF-based rehabilitation plan. All individuals were offered to participate in lectures and discussions about LEoP, rehabilitation methodology, how to manage an ICF-based rehabilitation plan, self-management strategies, and various interventions, for example, energy management, physical activity in relation to LEoP, and national laws and regulations relevant for people with disability. In addition, each team member provided tailored interventions based on their professional expertise and according to the rehabilitation plan, with the aim to meet each individual’s needs and to achieve the goals in the rehabilitation plan. For example, advice regarding exercises, home visits to discuss and plan home adaptations, provision of technical devices and orthopedical equipment, advice regarding mobility, mobility training, advice and practical skills training and strategies for managing everyday life activities, and different types of certificates for economic support, assistance or for sick leave, were provided. At the end of the rehabilitation program, goals were evaluated together by the individual and the team. A written discharge plan was made, and individual follow-ups were offered, depending on each individual’s needs and preferences.

### 2.5 The Canadian Occupational Performance Measure (COPM)

The COPM is an individualized, client-centered assessment tool, designed to capture an individual’s self-perceived performance and satisfaction with performance of daily activities (Law et al., 2014). The COPM covers three areas that each comprise three subgroups of activities: i) self-care (personal care, functional mobility, and community management), ii) productivity (paid/unpaid work, household management, and play/school), and iii) leisure (quiet recreation, active recreation, and socialization). The COPM interview starts with an open question where the individual identifies daily activities that he or she finds difficult to perform. Thereafter, the individual rates the importance of each of the identified activities on a 10-point Visual Analogue Scale (VAS), ranging from 1 (not at all important) to 10 (extremely important). Finally, the most important activities are prioritized. For these, performance and satisfaction with performance are rated on a similar VAS-scale (ranging from “not able to do” to “able to do extremely well” and “not satisfied” to “extremely satisfied”); higher ratings indicate greater importance, better performance, and more satisfaction.

The COPM is administered by the occupational therapist together with the individual, on admission to the rehabilitation program and at discharge from the program. Thereafter, the ratings for performance and satisfaction with performance are summarized into a mean score for performance and satisfaction. Thus, one mean score for performance and one mean score for satisfaction with performance are obtained for each individual each time the COPM is administered. When the COPM is used as an outcome measure, the difference between the mean scores at discharge and on admission for each individual constitutes a change score for performance and for satisfaction with performance. The COPM ratings are ordinal data, but according to the manual and in general practice, they are treated as continuous variables and therefore mean scores are calculated (Law et al., 2014).

### 2.6 Data and statistical analyses

The sociodemographic data were analysed descriptively and the number of prioritized activities within each COPM occupational area and subgroup were summarized for all participants.

We utilized the mean scores for performance and satisfaction with performance for all individuals, and with a paired-sample *t*-test, the change scores at a group level were analyzed. The effect size was used to calculate the change between the performance and satisfaction with performance mean scores, on admission compared to discharge, using Cohen’s d (0.2 = small, 0.5 = medium, 0.8 = large effect).

In 2011, updated thresholds for COPM change scores were presented (Eyssen et al., 2011); a change score greater than 1.4 points represents a clinically relevant change in performance and a change score greater than 1.9 points represents a clinically relevant change in satisfaction with performance. These thresholds have also been suggested in other studies (Karhula et al., 2022; Kos et al., 2016). In the present study, the thresholds were used to summarize how many participants that had change scores equal to or greater than 1.4 (performance) and 1.9 (satisfaction with performance), as well as those that had change scores that had not improved or had decreased following the rehabilitation program. Finally, the number of activities for each COPM area and subgroup that had improved at least 1.4 points (performance) and 1.9 points (satisfaction with performance) were calculated.

Pearson correlation coefficients were used to evaluate any relationships between the change scores for performance and satisfaction with performance, on admission and at discharge to the rehabilitation program.

Significance levels less than 5% are considered significant. All statistical analyses were performed with the SPSS version 25.0.

## 3 Results

### 3.1 Participants

In Table 1, data for the 102 participants are presented. They were 18 years or older, had a confirmed history of acute poliomyelitis with new symptoms after a period of functional stability of at least 15 years (clinically and electromyographically, EMG, verified). Their mean age was 61 (SD 9.7, range 29-81) years. Fifty percent of the men and 22% of the women were working. More than half of the participants used a mobility device and 30% used a power wheelchair/powered scooter. Most of the participants (93%) used some type of orthotic device.

**Table 1:** Characteristics of the 102 individuals with late effects of polio (LEoP)

	Men	Women
Sex, n (%)	28 (27%)	74 (73%)
Age, mean years (min-max)	58 (40–76)	62 (29–81)
Diagnosis
Age at acute poliomyelitis, mean years (min-max)	6 (1–20)	5 (1–18)
Mean years before post-polio onset, (min-max)	41 (20–60)	38 (1–70)
Country of birth, n (%)
Sweden	21 (8%)	63 (85%)
Nordic countries	2 (7%)	4 (5%)
Europe	2 (7%)	2 (3%)
Other countries	3 (11%)	5 (7%)
Living situation, n (%)
Living with another person (spouse, partner, children)	20 (71%)	47 (64%)^a^
Living alone	8 (29%)	26 (35%)^a^
Vocational situation, n (%)
Work	14 (50%)	16 (22%)^a^
Health insurance	6 (21%)	28 (38%)^a^
Old-age pension	7 (25%)	28 (38%)^a^
Other	1 (4%)	1 (2%)^a^
Accommodation, n (%)
House	13 (46%)	39 (53%)^a^
Apartment	15 (54%)	34 (46%)^a^
Assistive devices, n (%)
Wheelchair, manual	9 (32%)	7 (10%)
Wheelchair, power	2 (7%)	17 (23%)
Walker	8 (29%)	24 (32%)
Crutches	14 (50%)	20 (37%)
Orthopaedic technical aids	27 (96%)	66 (89%)
Personal care	11 (39%)	29 (39%)
Household	4 (14%)	21 (28%)

Note: ^a^ One missing value.

### 3.2 On admission to interdisciplinary rehabilitation

#### 3.2.1 Prioritized activities

The 102 participants prioritized 506 activities (mean 5; SD 9.7, range 1 to 22) that they found difficult to perform. The prioritized activities were in the area self-care (49%), followed by productivity (29%) and leisure (22%). None of the participants prioritized activities in the subgroup play/school, in the area productivity.

#### 3.2.2 Mean scores of performance and satisfaction with performance

In Table 2, data for performance and satisfaction with performance on admission are presented. The highest mean performance scores for all participants were in the subgroup socialization (5.9), followed by paid/unpaid work (5.8), whereas the lowest mean performance scores were found in the subgroup functional mobility (4.9). The highest mean scores of satisfaction with performance were found in socialization (6.0), and quiet recreation as the lowest (4.4).

**Table 2:** The number of prioritized activities (N = 506) that the 102 individuals with LEoP perceived as difficult to perform according to the COPM, including performance and satisfaction with performance on admission and at discharge

	Prioritized occupations	Performance			Satisfaction with performance		
	N (%)	On admission^a^	At discharge^a^	Number of	On admission^a^	At discharge^a^	Number of
				activities that			activities that
				improved			improved
				at least ≥1.4			at least≥1.9
				N (%)			points N (%)
Self-care	246 (49)			58 (24)			68 (28)
Personal care	67 (13)	5.0 (SD 2.2, 1–10)	5.7 (SD 2.3, 1–10)	16 (24)	4.6 (SD 2.9, 1–10)	5.3 (SD 2.7, 1–10)	21 (31)
Functional mobility	133 (26)	4.9 (SD 2.3, 1–10)	5.5 (SD 2.2, 1–10)	33 (25)	4.5 (SD 2.9, 1–10)	5.1 (SD 2.8, 1–10)	37 (28)
Community management	46 (9)	5.0 (SD 2.5, 1–10)	5.2 (SD 2.3, 1–10)	9 (20)	4.5 (SD 2.9, 1–10)	5.1 (SD 2.6, 1–10)	10 (22)
Productivity	147 (29)			31 (21)			40 (27)
Paid/ unpaid work	24 (5)	5.8 (SD 3.2, 1–10)	6.5 (SD 3.3, 1–10)	7 (29)	4.8 (SD 3.0, 1–10)	5.3 (SD 3.5, 1–10)	8 (33)
Household management	123 (24)	5.6 (SD 2.5, 1–10)	6.0 (SD 2.4, 1–10)	24 (20)	5.3 (SD 3.0, 1–10)	5.9 (SD 2.9,1–10)	32 (26)
Play/school	0	0	0	0	0	0	0
Leisure	113 (22)			20 (18)			23 (20)
Quiet recreation	31 (6)	5.2 (SD 3.2, 1–10)	5.3 (SD 2.7, 1–10)	8 (26)	4.4 (SD 3.4, 1–10)	5.6 (SD 2.8, 1–10)	10 (32)
Active recreation	66 (13)	5.0 (SD 2.7, 1–10)	5.5 (SD 2.6, 1–10)	11 (17)	5.3 (SD 3.2, 1–10)	5.7 (SD 3.2, 1–10)	10 (15)
Socialization	16 (3)	5.9 (SD 2.2, 1–10)	5.9 (SD 2.0, 1–9)	1 (6)	6.0 (SD 3.3, 1–10)	6.6 (SD 2.8, 2–10)	3 (19)

^a^Values are presented as mean score, SD and range; Note: Canadian Occupational Performance Measure (COPM).

### 3.3 At discharge from interdisciplinary rehabilitation

#### 3.3.1 Mean scores of performance and satisfaction with performance

The highest mean performance scores were found in the subgroup unpaid/paid work (6.5), and the lowest in the subgroup community management (5.2). For satisfaction with performance, the highest mean scores were found in socialization (6.6), and functional mobility and community management as the lowest (5.1) (cf. Table 2).

### 3.4 Change scores for performance and satisfaction with performance

There was a statistically significant increase in the mean performance scores between admission (5.18; SD 1.9) and discharge (5.66; SD 1.8) (*p* < 0.001). The mean increase in the performance change scores was 0.48 with a 95% confidence interval ranging from 0.28 to 0.68. For satisfaction with performance there was a statistically significant increase between mean scores from admission (mean 4.9; SD 2.2) to discharge (mean 5.4; SD 2.1) (*p* < 0.001). The mean increase in satisfaction with performance change scores was 0.50 with a 95% confidence interval ranging from 0.20 to 0.80. The effect size between the change scores was 0.20 (performance) and 0.22 (satisfaction with performance).

**Fig. 2:**
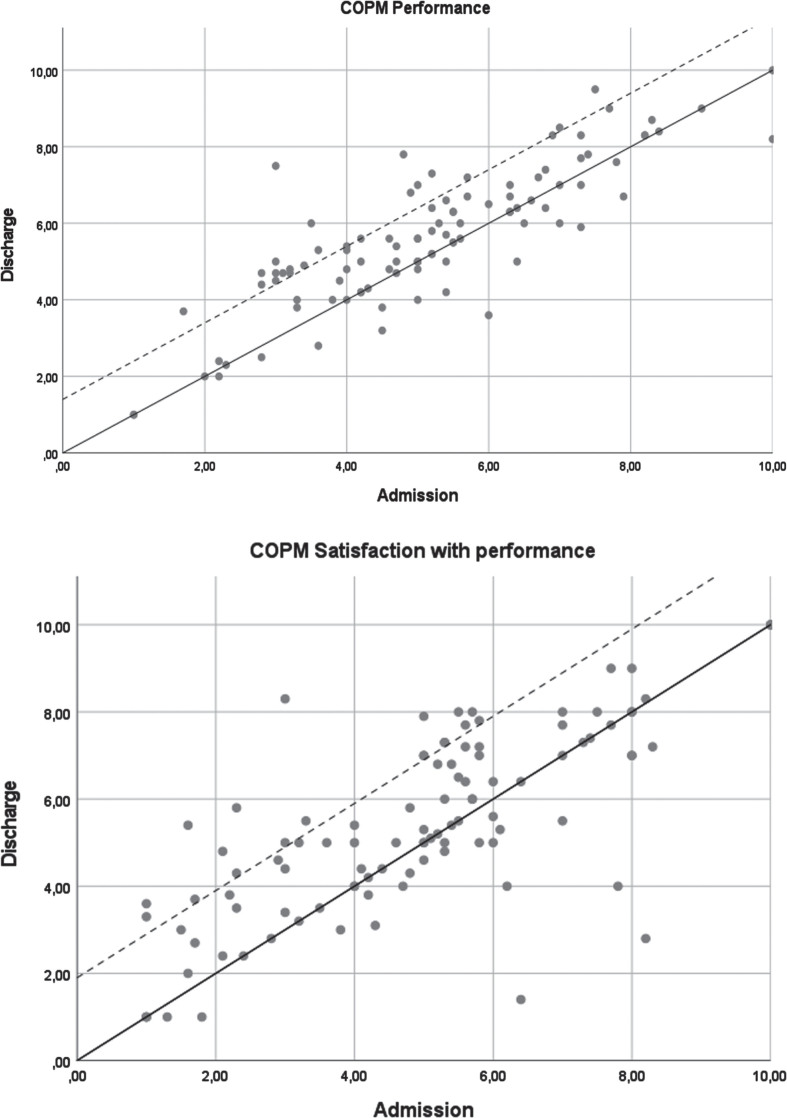
Each participant’s individual mean score on admission and at discharge.

In Fig. 2, the mean scores for performance and satisfaction with performance on admission and at discharge for each participant are plotted against each other. The dotted line indicates the cut-off for change scores of 1.4 or above for performance and change scores of 1.9 or above for satisfaction with performance (Eyssen et al., 2011). There was a statistically significant correlation between the change in performance and in satisfaction with performance from admission to discharge (*r* = 0.43; *p* < 0.00). No statistically significant correlation was found between performance on admission and the differences at discharge, or between satisfaction with performance on admission and the differences at discharge.

Twenty-three participants (22%) had change scores for performance that were equal to or greater than 1.4 points, whereas for 25 participants, no change had occurred between discharge and admission, and 19 participants had change scores that had decreased at discharge. Similar, 19 participants (19%) had change scores for satisfaction with performance that were equal to or greater than 1.9 points at discharge. For 27 participants, no change had occured between discharge and admission for satisfaction with performance, and 22 participants had change scores that had decreased at discharge. Six participants had change scores above the cut-off for both performance and satisfaction with performance.

#### 3.4.1 Number of activities where performance and satisfaction with performance had changed

At discharge, 174 (35%) of the 506 prioritized activities had increased performance scores, 250 (49%) were unchanged, and 74 (14%) had decreased scores compared to on admission. For satisfaction with performance, 131 (26%) activities had increased scores at discharge, 250 (49%) activities were unchanged, and 57 (11%) activities had decreased.

Most of the activities that showed an increase in mean scores for performance were in the subgroup paid/unpaid work (29 %), quiet recreation (26 %), and functional mobility (25 %) (cf. Table 2). Thirty-three percent of activities related to paid/unpaid work had improved their mean scores of satisfaction with performance, 32% for quiet recreation, and 31% for personal care.

## 4 Discussion

In this retrospective study, we evaluated changes in performance and satisfaction with performance of daily activities in 102 people with LEoP, following interdisciplinary rehabilitation. There was a statistically significant increase in the mean scores of performance (from 5.18 to 5.66) and satisfaction with performance (from 4.90 to 5.40) between admission and discharge. Increased change scores were found for 22% of the participants for performance (≥1.4 points), 19 % of the participants for satisfaction with performance (≥1.9 points), and six percent had increased their change scores for both scales. At the same time, we found no change for 25% of the participants for performance, and for 26% of the participants for satisfaction with performance. Further, a decrease in change scores were found for 19% (performance) and 22% (satisfaction with performance) of the participants.

The reported results are similar to those presented in other studies where rehabilitation interventions in people with LEoP were evaluated (Davidson et al., 2009; Curtis et al., 2020), but compared to these studies, our participants improved performance with daily activities to a larger extent. Our results are also in line with previous research where the COPM was used to evaluate change after rehabilitation interventions in people with Multiple Sclerosis (Kos et al., 2016; Månsson Lexell et al., 2014) and musculoskeletal pain (Persson et al., 2004). For instance, the statistically significant increase in the present study is based on improvements for more than half of the participants (57% for performance and 52% for satisfaction with performance). Månsson Lexell et al. (2014) and Kos et al. (2016), reported similar increases for performance (42% and 59% of the participants, respectively) as well as for satisfaction with performance (56% and 41% of the participants, respectively). Comparing our results to the two previous studies with people with LEoP, Davidson et al. (2009) reported 21% of their participants had increased both performance and satisfaction with performance compared to only 8% of our participants. Further, our participants had a mean change score of 0.48 for performance and 0.50 for satisfaction with performance, whereas Davidson et al. (2009) reported higher mean change scores for both performance (1.2) and satisfaction with performance (1.3). However, due to the large difference in sample size (24 vs 102), comparisons should be interpreted with caution. The 153 participants in the study by Curtis et al. (2020) showed no change for performance whereas satisfaction with performance had a mean change score of 0.6, which is slightly higher than the participants in our study. On the other hand, our participants had a larger mean change score for performance (0.48).

The intervention in the present study was interdisciplinary, goal-oriented and tailored to each participant. The rehabilitation process followed the same steps, but the goals in the rehabilitation plan determined the specific interventions. This is different from the participants in Davidson et al. (2009) who completed a program focusing on mobility problems where wheelchairs users were excluded, and many of the participants had previously received rehabilitation. It is therefore possible that this selected group of participants were better prepared to make use of the intervention and to implement a change. In contrast, the participants in our study had not received interdisciplinary rehabilitation before, thereby representing individuals with a variety of unmet needs. This may also explain why the Davidson participants had higher change scores compared to our participants. In addition, the Davidson program (Davidson et al., 2009) was mainly delivered as a group program whereas our program was individualized. Our rehabilitation program offered a broader repertoire of individually tailored interventions that, for example, focused on self-management, lifestyle changes and how to implement compensatory strategies and techniques. It is therefore similar to the one described by Curtis et al. (2020), which addressed aspects such as individual goal-setting, content that facilitated change, fatigue management, tailored exercise, diet recommendations, and sleep and respiratory considerations. Further, the results from our study, and those performed with people with MS (Kos et al., 2016; Månsson Lexell et al., 2014), are based on scores obtained on admission and at discharge, whereas the studies performed with people with LEoP (Davidson et al., 2009; Curtis et al., 2020) were based on scores collected on admission and at a six-month follow-up. Kos et al. (2016) added a three-month follow-up showing additional improvements for both performance (from 59% to 71% of the participants) and satisfaction with performance (from 41% participants to 50%). This suggests that longer-term follow-up may be motivated in order to capture benefits from rehabilitation interventions focusing on self-management perspectives.

Despite the significant increases in COPM scores between admission and discharge in the present study, improvements above the established thresholds of 1.4 (performance) and 1.9 (satisfaction with performance) were only seen in 22% and 19% of the participants, respectively. Furthermore, only six participants (6 %) had change scores above the cut-offs for both performance and satisfaction with performance. There are also participants who rated their performance (19%) and satisfaction with performance (22%) lower at discharge than on admission. Curtis et al. (2020) discussed that although their participants did not reach change scores above the established thresholds, they did not decline from baseline, arguing it was a positive sign when living with a chronic condition. However, other authors (Persson et al., 2004) suggested that a decline in performance and satisfaction with performance scores at discharge could be a result from the rehabilitation process itself which encourage participants to engage in, and deal with their activity limitations and participation restrictions they previously had difficulty to understand or accept. We also know from previous research (Månsson Lexell et al., 2015) that individuals with LEoP who participate in an interdisciplinary self-management rehabilitation program struggle to adapt to the consequences of their life situation. This is also consistent with other research (Larsson Lund & Lexell, 2010; Hagelskjaer et al., 2021), stating that self-mangement rehabilitation interventions often require longer implementation periods. Thus, it is reasonable to believe that if an additional follow-up assessment had been performed 6 to 12 months after program completion, similar to previous studies (Davidson et al., 2009; Curtis et al., 2020), our study participants could have had larger improvements in their COPM scores.

On admission, the participants included in our study reported a variety of activity problems (*n* = 506) connected to self-care (246; 49%), productivity (147; 29%), and leisure (113; 22%). This is consistent with previous studies that have described difficulties in activities among persons with LEoP (Appelin et al., 2014; Davidson et al., 2009), also showing that although the rehabilitation interventions were different in our study compared to those in Davidson et al. (2009), similar activity goals were targeted. Still, there is a lack of knowledge if rehabilitation outcomes are dependent on the individuals’ function, the severity of LEoP-related symptoms or other factors, an area for future research.

### 4.1 Methodological limitations

There are some limitations to this study. No control group was included due to the retrospective design. Another limitation is related to the use of the COPM as both a tool to identify problems with daily activities and as an outcome measure. In the present study we not only wanted to present mean scores of performance and satisfaction with performance but also more detailed information about the type of activities that were prioritized, which resulted in a large exclusion of participants. For instance, only scores for performance and satisfaction with performance were available for 106 individuals on admission and at discharge. Because detailed information on which activities were problematic were lacking, these individuals were excluded. Despite the large exclusion, this study included a fairly large number of participants. For another 74 individuals that were excluded, the COPM had only been administered on admission and had not been used as an outcome measure. It is possible that these individuals had become aware of, and altered daily life to such an extent that they no longer performed the same activities at discharge as on admission. We know from previous research (Sturkenboom et al., 2014; Wressle et al., 2002), that when individuals become aware of their limitations, some activities are no longer relevant for them to engage in. Thus, adding other outcome measures is therefore warranted in future studies.

### 4.2 Conclusion

This is one of the largest studies that has evaluated changes in performance and satisfaction with performance of daily activities among people with LEoP following interdisciplinary rehabilitation. The results imply that interdisciplinary rehabilitation can enhance self-rated performance and satisfaction with performance of daily activities among people with LEoP. Future studies of rehabilitation for people with LEoP should use a prospective design, capture the participants’ process of change related to their rehabilitation period and include longer follow-up periods.

## Declaration of interest

The authors have no competing interest to declare.

## Ethical considerations

The study has been approved by the Swedish Ethical Review Authority (2020–05313) and was performed in accordance with medical research involving human subjects as described in the Declaration of Helsinki.

## Informed consent

At the time of admission to interdisciplinary rehabilitation, each participant received information about the clinical database and provided written informed consent to have their data included.

## Funding

This study was funded by the Norrbacka-Eugenia Foundation and the Promobilia Foundation.

## References

[ref001] Ahlström, G. , Karlsson, U. (2000). Disability and quality of life in individuals with postpolio syndrome. Disability and Rehabilitation, 22(9), 416–422. 10.1080/096382800406031 10894205

[ref002] Appelin, K. , Lexell, J. , Lexell, E. M. (2014). Perspectives of occupations that people with late effects of polio perceive difficult to perform. Occupational therapy international,, 21(3), 98–107. 10.1002/oti.1368 24619836

[ref003] Bertelsen, M. , Broberg, S. , Madsen, E. (2009). Outcome of physiotherapy as part of a multidisciplinary rehabilitation in an unselected polio population with one-year follow-up: an uncontrolled study. Journal of Rehabilitation Medicine, 41(1), 85–87. 10.2340/16501977-0282 19197575

[ref004] Curtis, A. , Lee, J. S. , Kaltsakas, G. , Auyeund, V. , Shaw, S. , Hart, N. , Steier, J. (2020). The value of a post-polio syndrome self-management programme. Journal of Thoracic Disease, 12(Suppl 2), S153–S162. 10.21037/jtd-cus-2020-009 33214920 PMC7642628

[ref005] Davidson, A. C. , Auyeung, V. , Luff, R. , Holland, M. , Hodgkiss, A. , Weinman, J. (2009). Prolonged benefit in post-polio syndrome from comprehensive rehabilitation: A pilot study. Disability and Rehabilitation, 31(4), 309–317. 10.1080/09638280801973206 18608421

[ref007] Eyssen, I.C., Steultjens, M.P., Oud, T.A., Bolt, E.M., (2011). Responsiveness of the Canadian Occupational Performance Measure. Journal of Rehabilitation Research and Development, 48(5), 517–528. 10.1682/jrrd.2010.06.0110. 21674402

[ref008] Hagelskjaer, V. , Torma Nielsen, K. , von Bulow, C. , Gregersen Oestergaard, L. , Graff, M. , Ejlersen Waehrens, E. (2021). Evaluating a complex intervention addressing ability to perform activities of daily living among persons with chronic conditions: study protocol for a randomized controlled trial (ABLE). BMJ Open, 11, e051722. 10.1136/bmjopen-2021-051722 PMC8628341 34836902

[ref009] Jönsson, A-L. , Möller, A. , Grimby, G. (1999). Managing occupations in everyday life to achieve adaptation. American Journal of Occupational Therapy, 53(4), 353–362. 10427677

[ref010] Karhula, M.E. , Kanelisto, K. , Hämäläinen, P. , Ruutiainen, J. , Era, P. , Häkkinen, A. , Salminen, A-L. (2022). Self-reported Reasons for Changes in Performance of Daily Activities During a 2-year Multidisciplinary Multiple Sclerosis Rehabilitation. International Journal of MS Care, 24(3), 110–116. 10.7224/1537-2073.2020-061 35645629 PMC9135369

[ref011] Kling, C. , Persson, A. , Gardulf, A. (2002). The health-related quality of life of patients suffering from the late effects of polio (post-polio). Journal of Advanced Nursing, 32(1), 164–173. 10.1046/j.1365-2648.2000.01412.x 10886448

[ref012] Kos, D. , Duportail, M. , Meirte, J. , Meeus, M. , D’hooghe, M. , Nagels, G. , Willekens, B. , Meurrens, T. , Ilsbroukx, S. , Nijs J. (2016). The effectiveness of a self-management occupational therapy intervention on activity performance in individuals with multiple sclerosis-related fatigue: a randomized-controlled trial. International Journal of Rehabilitation Research, 39(3), 255–262. 10.1097/MRR.0000000000000178 27182847

[ref013] Körner, M. (2010). Interprofessional teamwork in medical rehabilitation: a comparison of multidisciplinary and interdisciplinary team approach. Clinical Rehabilitation,, 24(8), 745–755. 10.1177/0269215510367538 20530646

[ref014] Larsson-Lund, M. , Lexell, J. (2010). A positive turning point inlife – how persons with late effects of polio experience the influence of an interdisciplinary rehabilitation programme. Journal of Rehabilitation Medicine, 42(6), 559–565. 10.2340/16501977-0559 20549161

[ref015] Law, M. , Baptiste, S. , Carswell, A. , McColl, M.A. , Polatajko, H. , Pollock, N. (2014). Canadian Occupational Performance Measure (5th ed.). Ottawa, Canada: CAOT Publications ACE.

[ref016] Lexell, J. (2012). What’s on the Horizon: Defining Physiatry through Rehabilitation Methodology. Physical Medicine and Rehabilitation, 4(5), 331–334. 10.1016/j.pmrj.2012.03.009 22613361

[ref017] Lexell, J. , Brogårdh, C. (2015). The use of ICF in the neurorehabilitation process. Neuro Rehabilitation,, 36(1), 5–9. 10.3233/NRE-141184 25547759

[ref018] Lo, J.K. , Robinson, L.R. (2018). Post-polio syndrome and the late effects of poliomyelitis: Part 2. treatment, management and prognosis. Muscle & Nerve, 58(6), 760–769. 10.1002/mus.26167 29752826

[ref019] Lorig, K. , Holman, H. (2003). Self-management education: history, definition, outcomes, and mechanisms. Annals of Behavioral Medicine, 26(1), 1–7. 10.1207/S15324796ABM2601_01. 12867348

[ref020] McNalley, T. E. , Yorkston, K. M. , Jensen, M. P. , Truitt, A. R. , Schomer, K. G. , Baylor, C. , Molton, I. R. (2015). Review of secondary health conditions on postpolio syndrome. American Journal of Physical Medicine & Rehabilitation, 94(2), 139–145. 10.1097/PHM.0000000000000166 25122095 PMC4289114

[ref021] Månsson Lexell, E. , Flansbjer, U-B. , Lexell, J. (2014). Self-perceived performance and satisfaction with performance of daily activities in persons with multiple sclerosis following interdisciplinary rehabilitation. Disability and Rehabilitation, 36(5), 373–378. 10.3109/09638288.2013.797506 23735012

[ref022] Månsson Lexell, E. , Lexell, J. , Larsson Lund, M. (2015). Therehabilitation plan can support clients‘ active engagement and facilitate the process of change-experiences from people with lateeffect of polio participating in a rehabilitation programme. Disability and Rehabilitation, 38(4), 1–8. 10.3109/09638288.2015.1038363 25893398

[ref023] Persson, E. , Rivano-Fisher, M. , Eklund, M. (2004). Evaluation of changes in occupational performance among patients in a pain management program. Journal of Rehabilitation Medicine, 36(2), 85–91. 10.1080/16501970310019142 15180223

[ref024] Sjödahl Hammarlund, C. , Lexell, J. , Brogårdh, C. (2020). Self-reported impairments among people with late effects of polio: amixed-method study. Journal of Rehabilitation Medicine, 52(7). 10.2340/16501977-2706 32556343

[ref025] Sturkenboom, I.H., Graff, M.J., Hendriks, J.C., Veenhuizen, Y. , Munneke, M. , Bloem, B.R. , Nijhuis-van der Sanden, M.W., OTiP study group (2014). Efficacy of occupational therapy for patients with Parkinson’s disease: a randomised controlled trial. Lancet Neurology, 13(6), 557–66. 10.1016/S1474-4422(14)70055-9 24726066

[ref026] Thorén-Jönsson, A-L. (2001). Ability and perceived difficulty in daily activities in people with poliomyelitis sequelae. Journal of Rehabilitation Medicine, 33(1), 4–11. 10.1080/165019701300006461 11480469

[ref027] Townsend, E. , Polatajko, H. (2007). Enabling occupation II: Advancing an occupational therapy vision for health, well-being & justice through occupation. Ottawa: CAOT Publications ACE.

[ref028] Wade, D. (2015). Rehabilitation- a new approach. Overview and Part One: the problems. Clinical Rehabilitation, 29(11), 1041–1050. 10.1177/0269215515601174 26467940

[ref029] Wressle, E. , Marcusson, J. , Henriksson, C. (2002). Clinical utility of the Canadian Occupational Performance Measure- Swedish version. Canadian Journal of Occupational Therapy, 69(1), 40–48. 10.1177/000841740206900104 11852689

